# Neurocysticercosis in transition: expanding clinical spectrum, evolving diagnostics, and emerging therapies

**DOI:** 10.3389/fphar.2026.1748002

**Published:** 2026-02-19

**Authors:** Do-Youn Lee, Hima Bindu Mantravadi, Dinesh Puri, Amit Kumar Gupta, Preeti Dnyandeo Sonje, Sorabh Lakhanpal, Sujeet Kumar Singh, Sanjay Kumar, Karen Jaison

**Affiliations:** 1 College of General Education, Kookmin University, Seoul, Republic of Korea; 2 Department of Microbiology, Malla Reddy Institute of Medical Sciences, Hyderabad, Telangana, India; 3 Faculty of Pharmaceutical Sciences, Graphic Era Hill University, Dehradun, India; 4 Centre for Promotion of Research, Graphic Era Deemed University, Dehradun, India; 5 Department of Radiology, School of Medical Sciences and Research, Sharda University, Greater Noida, India; 6 Department of Anatomy, Dr. D. Y. Patil Medical College, Hospital and Research Centre, Dr. D. Y. Patil Vidyapeeth (Deemed-to-be-University), Pune, Maharashtra, India; 7 School of Pharmaceutical Sciences, Lovely Professional University, Phagwara, India; 8 Department of Biotechnology, Noida Institute of Engineering and Technology (Pharmacy Institute), Greater Noida, India; 9 Centre for Research Impact and Outcome, Chitkara College of Pharmacy, Chitkara University, Rajpura, Punjab, India; 10 Saveetha Medical College and Hospital, Saveetha Institute of Medical and Technical Sciences, Saveetha University, Chennai, India

**Keywords:** clinical spectrum, epilepsy, neurocysticercosis, seizures, Taenia solium

## Abstract

Neurocysticercosis (NCC) infection of the central nervous system by *Taenia solium* larvae, remains a leading cause of acquired epilepsy in endemic regions and an increasingly recognized imported disease elsewhere. The traditional view of NCC as a solitary parenchymal cyst causing seizures has shifted to a heterogeneous syndrome shaped by parasite burden, stage, location, and host immune response. Clinical manifestations extend beyond seizures to headaches, cognitive impairment, psychiatric symptoms, visual loss, movement disorders, and stroke. Progress in neuroimaging, serology, and molecular diagnostics has improved case detection and disease phenotyping, while management increasingly relies on stage and compartment specific combinations of antiparasitic drugs, anti-inflammatory therapy, and neurosurgical or endoscopic interventions for extraparenchymal disease and hydrocephalus. Persistent gaps include limited randomized evidence, incomplete validation of diagnostic algorithms, and constrained access to advanced care in high-burden regions, underscoring the need for coordinated research and implementation strategies to reduce NCC’s global neurological impact.

## Introduction

Neurocysticercosis (NCC) is the most important parasitic infection of the human central nervous system and a leading cause of adult-onset seizures and epilepsy in many low- and middle-income countries where the parasite is endemic (Garcia et al., 2014; Gripper and Welburn, 2017). It results from CNS infection with the larval stage of *Taenia solium*, a zoonosis tightly linked to poverty, free-roaming pigs, poor sanitation, and inadequate meat inspection (Garcia et al., 2014; Mwape et al., 2013). Human infection follows ingestion *of T. solium* eggs, with larvae encysting in brain parenchyma, ventricles, subarachnoid spaces, or ocular structures. Over the past 2 decades, however, the field has undergone a fundamental transition. NCC is now recognized as a heterogeneous neuroinflammatory disease encompassing multiple well-defined clinical syndromes, shaped by parasite location, developmental stage, parasite burden, and host immune response. The clinical framework has evolved from a seizure-centric model to a syndrome-based approach that includes extraparenchymal disease, neuropsychiatric syndromes, movement disorders, hydrocephalus, cognitive impairment, and visual loss (Mahale et al., 2015; Singh et al., 2024). The diagnostic strategies have expanded beyond conventional neuroimaging to include refined serological assays, point-of-care antigen tests, and molecular tools, enabling earlier and more precise case identification. At the same time, treatment paradigms have shifted from uniform antiparasitic therapy to individualized regimens that integrate antiparasitic drugs, staged corticosteroid strategies, and neurosurgical or endoscopic interventions when indicated.

## Epidemiology

NCC is highly endemic across Latin America, sub-Saharan Africa, and parts of South and Southeast Asia, where human tapeworm carriers, environmental egg contamination, and pig exposure coexist (Garcia et al., 2014; Mahale et al., 2015). Community-based studies in endemic areas suggest that *T. solium* cysticercosis may affect 10%–20% of the population serologically, with a substantial fraction of active or calcified brain lesions detectable on CT (Gripper and Welburn, 2017). In some rural regions of Latin America and India, NCC is estimated to account for up to one-third of cases of adult-onset epilepsy (Garcia et al., 2014; Mahale et al., 2015). Beyond traditional endemic zones, NCC has become an important globalized infection. Increased migration, travel, and globalized food chains have led to rising numbers of cases in North America and Europe, often in migrants or their close contacts (Gripper and Welburn, 2017). Autochthonous transmission has been documented in several high-income countries when a tapeworm carrier contaminates food, as illustrated by a school outbreak in Belgium (Vanden Driessche et al., 2024). These events show that NCC is possible wherever basic sanitation and carrier detection fail, regardless of national income level.

From a neurological standpoint, NCC is now recognized as a major cause of epilepsy burden. A large cohort analysis confirmed a strong association between calcified NCC and mesial temporal lobe epilepsy with hippocampal sclerosis, emphasizing its role in chronic epileptogenesis (Secchi et al., 2021). In endemic areas, NCC also contributes to headache disorders, cognitive impairment, and stroke, particularly in subarachnoid and intraventricular forms (Mahale et al., 2015; Vasquez-Baiocchi et al., 2025).

## Life cycle of *Taenia solium*

**FIGURE 1:**
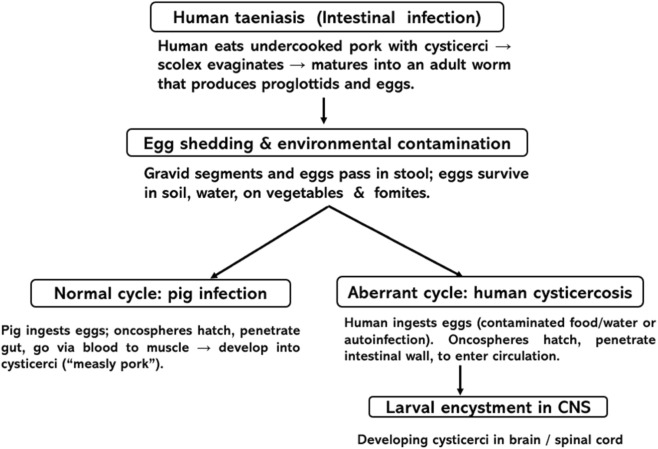
Schematic representation of the life cycle of neurocysticercosis.

Humans acquire taeniasis by ingesting cysticerci in undercooked pork, becoming definitive hosts harbouring adult tapeworms in the small intestine, which shed eggs and proglottids into the environment. Pigs, the normal intermediate host, ingest eggs and develop muscular cysticerci, completing the pig–human cycle. Neurocysticercosis arises when humans instead ingest *T. solium* eggs (contaminated food, water, or via autoinfection), acting as aberrant intermediate hosts. In the small intestine, the scolex evaginates, attaches to the mucosa, and matures into an adult tapeworm that sheds gravid proglottids and eggs in feces, contaminating soil, water, and food. Pigs ingest these eggs, the oncospheres hatch, penetrate the gut wall, and travel via the bloodstream to muscle, where they encyst as cysticerci (“measly pork”), completing the classical pig–human cycle. NCC emerges when humans instead ingest eggs (contaminated food/water or autoinfection in a tapeworm carrier), becoming aberrant intermediate hosts (Figure 1). Oncospheres hatch in the human intestine, penetrate the mucosa, and disseminate hematogenously to the brain, spinal cord, eye, and other tissues, producing systemic cysticercosis and, when the CNS is involved, resulting neurocysticercosis (Garcia et al., 2014; Gripper and Welburn, 2017).

## Pathophysiology and clinical spectrum

The clinical and radiologic course is largely determined by the stage of the cyst and the intensity of the host immune response. Within the CNS, larvae encyst and develop into cysticerci in different compartments: brain parenchyma, subarachnoid spaces, ventricular system, spinal canal, and ocular structures. In parenchymal NCC, cysts evolve through four main stages: vesicular, colloidal, granular–nodular and calcified (Table 1).

**TABLE 1:** Pathophysiology, stages and clinical presentation of neurocysticercosis.

Stage/Level	Underlying pathology	Key mechanisms	Clinical consequences
Cyst development (vesicular stage)	Oncospheres develop into viable cysticerci with scolex; cyst wall intact (Garcia et al., 2014)	Parasite secretes immunomodulatory molecules → local immune suppression; minimal inflammation	Often asymptomatic or mild headaches; seizures may occur if multiple or strategically located cysts
Host immune recognition (colloidal stage)	Parasite begins to degenerate; antigens leak from cyst (Prodjinotho et al., 2020)	Breakdown of immune evasion → strong inflammatory response: lymphocytes, macrophages, eosinophils, microglial activation; cytokines (IL-1β, TNF-α, IL-6) and Th2 cytokines around cyst	Perilesional edema, raised intracranial pressure, acute symptomatic seizures, focal deficits, headaches
Granuloma formation (granular–nodular stage)	Cyst collapses; contents resorbed; fibrous wall forms (Secchi et al., 2021)	Granulomatous reaction with epithelioid cells, giant cells, fibrosis; reduced but persistent inflammation	Transient enhancing lesion on imaging; seizures still possible but less frequent; symptoms often improve
Calcification (inactive stage)	Residual granuloma becomes calcified nodule (Secchi et al., 2021)	Mineral deposition (calcium salts) in fibrotic lesion; parasite dead. Intermittent perilesional edema can occur due to immune reactivation against residual antigens	“Inactive” on imaging, but can still cause chronic epilepsy, migraine-like headaches, and occasionally focal deficits
Extraparenchymal/racemose disease	Cysts in subarachnoid cisterns, basal cisterns, Sylvian fissure; often large, lobulated (“racemose”) (Xu et al., 2025)	Space-occupying multicystic masses; chronic meningitis; fibrosis of subarachnoid spaces; obstruction of CSF pathways	Hydrocephalus, raised ICP, cranial neuropathies, gait disturbance, cognitive changes; higher morbidity and mortality
Intraventricular disease	Cysts in lateral, third, or fourth ventricles, sometimes free-floating (Vasquez-Baiocchi et al., 2025)	Mechanical obstruction of CSF flow; ball-valve mechanism; ependymitis; inflammatory debris	Acute or intermittent obstructive hydrocephalus, headache, vomiting, papilledema, sudden death if acute blockage
Spinal and meningeal involvement	Cysts in spinal subarachnoid space or cord; chronic arachnoiditis (Garg et al., 2025b)	Inflammatory fibrosis, nerve root and cord compression, venous congestion	Radiculopathy, myelopathy, spastic paraparesis, bladder dysfunction
Ocular involvement	Cysts in vitreous, subretinal space, or orbit (Garg et al., 2025a)	Local inflammatory reaction; traction on retina; vitreitis	Blurred vision, floaters, retinal detachment, vision loss; may worsen with antiparasitic treatment if not removed
Vascular involvement (cysticercotic vasculitis)	Inflammation around penetrating arteries and arterioles (Mahale et al., 2015)	Necrotizing or proliferative angiitis, intimal thickening, thrombosis	Ischemic strokes, transient ischemic attacks, focal deficits
Seizure and epilepsy mechanisms	Both active and calcified lesions can trigger seizures (Secchi et al., 2021)	Cortical irritation by edema/inflammation; gliosis and network reorganization; association with mesial temporal sclerosis in some patients	Acute symptomatic seizures (active lesions) and chronic epilepsy (especially with calcifications/MTLE)
Immune modulation/chronicity	Long-term persistence of parasite or antigens in CNS (Perez-Osorio et al., 2025)	Parasite-induced immune downregulation (e.g., T-cell exhaustion, regulatory T cells, Th2 skewing) vs. damaging host inflammatory bursts	Chronic, relapsing course with episodes of edema, seizures, headaches; variable response to therapy

In the vesicular (viable) stage, cysticerci are thin-walled, fluid-filled vesicles with a visible scolex, surrounded by minimal inflammation. The parasite actively down-modulates local immunity, favouring regulatory and anti-inflammatory pathways and limiting oedema and gliosis (Prodjinotho et al., 2020). Many patients remain asymptomatic, but seizures or headaches can occur when lesions are numerous or cortically located. On imaging, these appear as CSF like cysts with the classic “hole-with-dot” sign. As the parasite begins to degenerate, the lesion enters the colloidal stage, which is the most pathogenic phase. Cyst wall breakdown and antigen leakage trigger a vigorous inflammatory reaction with activation of microglia, macrophages, and T cells, and production of pro-inflammatory cytokines such as TNF-α, IL-1β, and IL-6 (Miltenberger et al., 2025). The blood–brain barrier becomes disrupted, leading to marked perilesional oedema and mass effect. Clinically, this corresponds to acute symptomatic seizures, severe headache, focal deficits, and signs of raised intracranial pressure. CT and MRI typically show ring-enhancing lesions with extensive oedema (Garcia et al., 2014).

With time, inflammation becomes organised and the lesion transitions to the granular–nodular stage. The cyst collapses and is replaced by a smaller fibrotic granuloma; oedema recedes and enhancement diminishes. Symptoms often improve, but seizures may persist as networks reorganise around the lesional scar (Secchi et al., 2021). Ultimately, the lesion becomes a calcified nodule, representing an “inactive” but often epileptogenic stage.

In extraparenchymal NCC, disease is driven by inflammation and obstruction of cerebrospinal fluid spaces. Subarachnoid (racemose) NCC involves large, multiloculated cysts in the basal cisterns and fissures, causing chronic eosinophilic meningitis, arachnoiditis, and fibrosis, which result in hydrocephalus, cranial neuropathies, and inflammatory vasculitis with increased stroke risk. Intraventricular NCC causes mechanical obstruction of CSF flow and ependymitis, resulting in intermittent or acute obstructive hydrocephalus and potential sudden deterioration (Xu et al., 2025).

The same principles apply in ocular and spinal disease, where cysts in tight, functionally critical spaces provoke disproportionate damage through local inflammation and mass effect. Ocular cysts can cause vitreitis and retinal detachment; killing an intraocular cyst without prior surgical removal can trigger catastrophic inflammation and vision loss (Garg et al., 2025a). Spinal cysts may cause arachnoiditis, cord compression, and progressive myelopathy. Systematic reviews show NCC as a reversible cause of parkinsonism, chorea, dystonia, and other hyperkinetic syndromes, as well as significant vision loss from ocular and neuro-ophthalmic involvement (Garg et al., 2025a; Garg et al., 2025b).

Overall, NCC pathophysiology reflects a dynamic interplay between parasite stage and host immunity, evolving from immune evasion and silent infection to intense inflammatory injury and, finally, to a scarred but persistently epileptogenic CNS (Cangalaya et al., 2017; Torib et al., 2024). Frontal lobe NCC presenting predominantly with schizoaffective-like symptoms in an adolescent immigrant illustrates how lesions can masquerade as primary psychiatric disease, with improvement following antiparasitic therapy and antipsychotics (Annor et al., 2025). Racemose and intraventricular forms often present with hydrocephalus, cranial neuropathies, and complex CSF-flow disturbances, demanding combined neurosurgical–medical care (Mahale et al., 2015; Xu et al., 2025). These findings support a shift from viewing NCC purely as a seizure disorder toward a broader “neurocysticercosis syndrome” encompassing epilepsy, neuropsychiatric, ocular, motor, and cognitive domains.

Although NCC predominantly involves the central nervous system, T. solium larvae may also affect subcutaneous tissue and skeletal muscle. Subcutaneous cysticercosis typically presents as painless nodules, while muscular involvement may be asymptomatic or cause myalgia or pseudohypertrophy during cyst degeneration (Del Brutto, 2012). Imaging and serology aid diagnosis, and recognition of these extracranial forms supports evaluation for concurrent NCC and guides management decisions.

## Emerging diagnostics

### Imaging and treatment response

The diagnosis of neurocysticercosis (NCC) increasingly relies on a stepwise, context-sensitive diagnostic pathway that integrates clinical assessment with imaging, serological, and molecular tools. Neuroimaging remains the cornerstone of diagnosis. In high-resource settings, contrast-enhanced magnetic resonance imaging (MRI) is preferred, as it allows accurate characterization of cyst stage, burden, and anatomical compartment, with advanced sequences (e.g., FIESTA or 3D SPACE) improving detection of intraventricular and subarachnoid disease (Knott et al., 2025). Computed tomography (CT) remains a pragmatic first-line investigation in low-resource or endemic settings, particularly for identifying calcified lesions and acute hydrocephalus. Parenchymal lesions show a characteristic evolution from vesicular (thin-walled CSF-like cyst with scolex, little edema) to colloidal (ring enhancement, marked edema), granular nodular (shrinking, thick wall, less edema), and finally calcified nodules (Ratcliffe et al., 2023; Tellez-Arellano et al., 2025). After antiparasitic therapy, viable cysts typically shrink, lose enhancement, and calcify, though there may be transient worsening of edema, enhancement early on and persistent MRI abnormalities in subarachnoid and ventricular cases despite clinical cure (Sanchez Boluarte et al., 2025).

### Serology and point-of-care diagnostics

Serological testing complements imaging, especially when radiological findings are equivocal. Enzyme-linked immunoelectrotransfer blot (EITB) IgG assays offer high specificity, while circulating antigen detection better reflects viable infection and treatment response (Garcia et al., 2014). Importantly, rapid point-of-care antigen tests are emerging as valuable screening tools in endemic regions, facilitating early case identification and referral where advanced imaging is limited (Zulu et al., 2024). Lema et al. (2025) examined antigen ELISA and Western blot IgG for NCC in people living with HIV in Tanzania and found Western blot to be more sensitive, though overall performance was reduced in comparison to immunocompetent populations (Lema et al., 2025). They also explored sequential versus parallel testing strategies, finding that parallel testing maximized sensitivity, an important consideration for screening high-risk populations. Point-of-care (POC) tests based on cysticercus antibodies offer potential for field-friendly triage in resource-limited settings. POC test was evaluated in rural southern Tanzania and reported modest overall sensitivity for CT-confirmed NCC but high sensitivity for active vesicular lesions, suggesting that POC testing can effectively identify patients warranting referral for imaging (Stelzle et al., 2024).

### Molecular diagnostics and next-generation sequencing

Molecular diagnostics, including PCR-based assays and next-generation sequencing of cerebrospinal fluid, are reserved for diagnostically challenging cases, atypical presentations, or immunocompromised patients, primarily in high-resource settings (Wilson et al., 2019). Case-based reports, including ventricular and posterior fossa disease, illustrate how NGS can provide definitive etiologic confirmation when biopsy is risky or non-diagnostic (Azmi et al., 2025; Guanru et al., 2025). Deploying these modalities within a structured diagnostic pathway allows clinicians to tailor investigations to available resources while improving diagnostic accuracy and clinical decision-making.

### Differential diagnosis

The differential diagnosis of neurocysticercosis includes intracranial tuberculomas, toxoplasmosis, brain metastases, gliomas, and fungal granulomas. Distinction relies on lesion morphology, enhancement patterns, serology, epidemiologic exposure, and therapeutic response, as misdiagnosis may lead to inappropriate treatment (Del Brutto, 2012; Garcia et al., 2018).

## Management: medical and neurosurgical

### Antiparasitic therapy

Antiparasitic therapy for NCC is primarily based on albendazole and praziquantel, used alone or in combination, and is recommended for most patients with viable parenchymal cysts who lack contraindications such as uncontrolled intracranial hypertension (Cangalaya et al., 2017; Mahamat et al., 2015). Albendazole is administered at 15 mg/kg/day (maximum 800 mg/day) for 7–14 days for viable parenchymal NCC, acting by inhibiting parasite microtubule formation. Praziquantel, dosed at 50–100 mg/kg/day divided into three doses for 10–14 days, increases parasite membrane permeability to calcium, inducing paralysis and death (Garcia et al., 2018). Antiparasitic treatment is not routinely indicated for patients with only calcified lesions or when severe edema, mass effect, or hydrocephalus would make inflammatory worsening dangerous. Because therapy provokes an inflammatory response, corticosteroids are co-administered to reduce edema and prevent symptom exacerbation. Dexamethasone (0.1–0.2 mg/kg/day) or prednisone (1 mg/kg/day) is typically initiated prior to cysticidal treatment and continued for 1–2 weeks in uncomplicated parenchymal disease (Carpio and Romo, 2016). Targeted immunomodulatory therapies, particularly TNF-α inhibitors such as etanercept, have emerged as promising adjuncts in refractory or complicated NCC by attenuating excessive inflammatory responses while permitting effective cysticidal activity of antiparasitic agents such as albendazole and praziquantel (Fujiwara et al., 2017).

### Management of extraparenchymal, ocular, and spinal NCC

Extraparenchymal NCC carries the highest morbidity and requires phenotype-specific management. Red flags for extraparenchymal disease include signs of raised intracranial pressure, chronic meningitis, hydrocephalus, stroke-like episodes, or progressive visual loss. Intraventricular NCC frequently presents with obstructive hydrocephalus and warrants prompt neuroimaging with MRI using CSF-sensitive sequences, followed by endoscopic cyst removal and cerebrospinal fluid diversion when indicated, rather than upfront antiparasitic therapy (White et al., 2018). Subarachnoid or racemose NCC is characterized by sustained cytokine-mediated inflammation, arachnoiditis, and vasculitis, often necessitating prolonged corticosteroid therapy, cautious antiparasitic use, and selective neurosurgical intervention (Garcia et al., 2018). Ocular NCC constitutes a medical emergency, as intraocular cysts must be surgically removed before initiating antiparasitic treatment, since drug-induced cyst degeneration can precipitate severe inflammation and irreversible vision loss (Del Brutto, 2012). Spinal NCC, although rare, should be suspected in patients with progressive radiculopathy or myelopathy; magnetic resonance imaging is diagnostic, and surgical decompression is indicated in cases of significant cord or nerve root compression, followed by adjunctive medical therapy when appropriate (Carpio and Romo, 2016).

### Prevention of NCC

Prevention of neurocysticercosis relies on interrupting Taenia solium transmission through improved sanitation, hand hygiene, and safe water access. Control of pig farming practices, meat inspection, and treatment of human tapeworm carriers, combined with community education and One Health strategies, are essential to reduce disease incidence in endemic regions.

### Implementation challenges and resource gaps

Despite advances in NCC care, endemic regions face major implementation gaps due to limited access to imaging, diagnostics, and neurosurgical expertise, leading to delayed or empirical management. Expanding point-of-care diagnostics, strengthening referral systems, and integrating NCC care into epilepsy and public health programs are essential to reduce disease-related morbidity.

## Conclusion

NCC is undergoing a fundamental transition from a seizure-focused parasitic infection to a spectrum of well-characterized neuroinflammatory syndromes encompassing epilepsy, cognitive impairment, psychiatric manifestations, movement disorders, hydrocephalus, and visual loss. Advances in diagnostics, deeper understanding of host–parasite immunology and evolving pharmacologic and surgical strategies have transformed clinical care, while also revealing persistent evidence and implementation gaps. By adopting a phenotype-driven and context-sensitive approach, clinicians can improve diagnostic precision, tailor therapy to disease compartment and inflammatory risk, and reduce preventable complications. Embracing this transition perspective in both endemic and non-endemic settings offers a pathway toward improved patient outcomes and reduced global neurological disability from NCC.
